# Influence of Gamma Irradiation and Antioxidants on the Quality of Spicy Chicken Meat During Refrigerated Storage

**DOI:** 10.1002/fsn3.70940

**Published:** 2025-09-17

**Authors:** Guolin Li, Ping Lin, Yangbo He, Zhihui Liu, Yongfu Li

**Affiliations:** ^1^ Integrated Agricultural Development Research Institute Guizhou Provincial Academy of Agricultural Sciences Guiyang China; ^2^ Guizhou Jinnong Irradiation Technology co., Ltd Guiyang China

**Keywords:** antioxidants, chicken meat, gamma irradiation, quality

## Abstract

The effects of antioxidants on microbial quality, color, lipid oxidation, fatty acids, odor, and volatile compounds in irradiated spicy chicken were investigated. Chicken meat was treated with antioxidants (α‐tocopherol, phytic acid, tea polyphenols, and tertiary butydroquinone) and 4 kGy gamma irradiation. The results showed that the total viable bacteria (TVB) and total coliform count (TCC) were significantly decreased after irradiation, and the combination of four kinds of antioxidants had no direct antibacterial effect. However, the antioxidants significantly inhibited the color fading of chili oil and repressed the increase in the peroxide value (POV) in irradiated samples. The contents of monounsaturated fatty acids (MUFAs) and polyunsaturated fatty acids (PUFAs) in irradiated meat with antioxidants were significantly higher than those in samples without antioxidants. The E‐nose results indicate that the odor of the sample treated with irradiation and antioxidants was closer to that of the nonirradiated sample; in contrast, irradiated meat without antioxidants was distinguished from the others. Irradiation decreased the alcohols and esters, increased the alkenes, and induced the formation of 1,3‐bis(1,1‐dimethylethyl)‐benzene. Alkenes were abundant in irradiated meat with antioxidants after storage for 60 days. These results suggest that 4 kGy gamma irradiation combined with antioxidants would be an alternative way to control the negative effects during spicy chicken irradiation processing.

## Graphical abstract

Four antioxidants can inhibit color fading in 4 kGy irradiated spicy chicken and delay lipid oxidation in irradiated spicy chicken. Four antioxidants inhibited the degradation of MUFAs and PUFAs caused by irradiation as well as improve quality of spicy chicken.

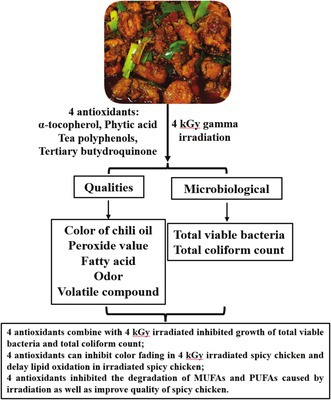

## 1 Introduction

Spicy chicken is a special local food in Guizhou Province of China that is made of chicken and red pepper as the main raw materials and fried with garlic, pepper, and other auxiliary materials. The meat industry has grown substantially in recent years due to the increasing demand for meat products and their excellent nutritional properties (Jia et al. 2021). Chicken meat is rich in nutrients, with the characteristics of high protein and low fat, and it also contains essential amino acids, minerals, vitamins, and relatively high levels of polyunsaturated fatty acids (Hassanzadeh et al. 2017). However, chicken meat is susceptible to microbial contamination owing to its abundant nutrients, high moisture content, and appropriate pH (Nerín et al. 2016; Verma et al. 2015). Traditional high‐temperature and high‐pressure processing easily results in negative effects, such as damaged appearance, texture, and flavor, as well as lower economic value (Verma et al. 2015; Huang and Ahn 2019). Gamma irradiation, considered a nonthermal preservation method, has been proven to control microorganisms, eliminate parasites, and extend shelf life in many different kinds of meat processing industries, including chicken meat, minced chicken meat (Abdeldaiem 2014), beef (Chen et al. 2007), camel meat (Al‐Bachir and Zeinou 2009), and goat meat (Jia et al. 2021). The World Health Organization (WHO) confirmed that an irradiation dose of no more than 10 kGy is generally considered not to change the safety of any food (Ravindran and Jaiswal 2019).

Irradiation technology could improve the quality and safety of meat products; moreover, some negative effects have emerged, including color change, odor change, and lipid oxidation, which are the vital factors that consumers use to judge meat quality (Huang and Ahn 2019; Yim et al. 2015). Although the meat body can generate some antioxidant substances like superoxide dismutase, catalase, peroxidase, glutathione peroxidase, glutathione reductase, and non‐enzymatic antioxidant compounds (Huang and Ahn 2019). However, using antioxidants is still an effective method to improve the irradiation preservation effect, reduce lipid oxidation, and extend the shelf life of meat (Xiao et al. 2011). Natural and synthetic antioxidants are usually applied to restrain the oxidative reactions developed during the processing of meat and meat products (Jayathilakan and Sultana 2018). α‐Tocopherol (vitamin E), phytic acid, tea polyphenols, and tertiary butylhydroquinone (TBHQ) are common antioxidants and have been approved for application as food additives in the meat processing industry in China according to the National standards of the People's Republic of China GB 2760‐2014.

Our previous studies found that low‐dose irradiation (2–4 kGy) could kill microorganisms and maintain meat quality, but the only problem was that the color of chili oil in spicy chicken fades (Li et al. 2023), mainly because capsanthin is very sensitive to irradiation (Ding et al. 2015). The underlying mechanism of capsanthin fading was direct oxidation or addition reactions in the presence of reactive oxygen species (ROS), which were generated by water molecules and lipid oxidation after irradiation (Nam et al. 2016; Jayathilakan and Sultana 2018).

The present study was designed to evaluate the effect of spicy chicken meat treatment with ^60^Co gamma irradiation combined with four kinds of antioxidants on improving meat quality during storage at a refrigerated temperature (4°C ± 2) to investigate the microbiological, color, lipid oxidation, fatty acid, and odor changes of spicy chicken meat during storage.

## 2 Materials and Methods

### 2.1 Materials

Spicy chicken meat was provided by Guizhou Longshan Xiangfang Food Co. Ltd. Spicy chicken is mainly made of fresh chicken (whole carcass), vegetable oil, and chili, and is fried. The spicy chicken was vacuum‐packed in polyethylene bags. Each bag contained 100 g of chicken meat in triplicate. All antioxidants were directly added to spicy chicken and mixed evenly.

### 2.2 Irradiation Treatment

All samples except for the control group were sealed in corrugated cases for ^60^Coγ irradiation at room temperature (18°C ± 2°C, RH 70%–75%), which was performed at Guizhou Jinnong Irradiation Technology Co. Ltd. The average dose rate of irradiation was 320 Gy/h. Samples were immediately stored at a cool temperature (4°C ± 1°C) after irradiation for further analysis. The actual absorbed dose was determined by the method of JJG 1028 to 91 (standard method for using the solver dichromate dosimeter to measure γ‐ray absorbed dose in water). The dose measuring bottles were placed in the front, rear, and center of the corrugated case.

### 2.3 Microbiological

The microbial counts of the total viable bacteria (TVB), the total coliform count (TCC), *Salmonella* spp., *Listeria monocytogenes* , and *Staphylococcus aureus* were determined, which were evaluated according to the methods of People's Republic of China GB 4789.2‐2016e, 4789.3‐2016f, GB 4789.4‐2016g, GB 4789.30‐ 2016c, and GB 4789.10‐2016d, respectively.

### 2.4 Color Determination

The color values were determined on the surface of spicy chicken packages by using a high‐quality colorimeter NR 200 (3NH Technology Co. Ltd., Shenzhen, China). The experiment was repeated three times, and each replicate was tested in three bags. Each experiment was performed three times. The experiment was performed after 0, 15, 30, 45, and 60 days of irradiation treatment. The standard white plate (*L* = 90.60, *a* = 0.23, *b* = −4.02) was used for calibration. The color difference (ΔE*) was calculated from the following equation:

$$\Delta E^{*}=\sqrt{{\left(\Delta L^{*}\right)}^{2}+{\left(\Delta a^{*}\right)}^{2}+{\left(\Delta b^{*}\right)}^{2}}$$

### 2.5 Peroxide Value

The peroxide value (POV) was assayed according to GB 5009.227‐2016b. Sixty grams of chicken meat from three bags was ground in a blender, 120 mL petroleum ether (boiling range: 30°C–60°C) was added, shaken well, fully mixed, allowed to stand and extract for 12 h, and filtered through a funnel containing anhydrous sodium sulfate. The filtrate was taken, petroleum ether was evaporated under reduced pressure with a rotary evaporator in a 40°C water bath, and the residue was tested. A total of 2.0 g of sample was accurately weighed, placed into a 250 mL iodine measuring bottle, added to a 30 mL chloroform glacial acetic acid mixture, and shaken gently to completely dissolve the sample. A total of 1.00 mL of saturated potassium iodide solution was added to the sample, the bottle was capped, and the mixture was gently shaken for 0.5 min and then placed in the dark for 3 min. One hundred milliliters of water was removed, shaken well, and immediately titrated with sodium thiosulfate standard solution (0.002 mol/L). When titrating to light yellow, 1 mL of starch indicator was added, and titrating was continued and shaken strongly until the blue color of the solution disappeared as the endpoint.

### 2.6 Fatty Acid Determination

The fatty acid was determined according to GB 5009.168‐2016a. Two grams of meat were accurately weighed; 100 mg of pyrogallic acid, a few zeolites, and 2 mL of 95% ethanol were added; and the mixture was mixed well. A total of 10 mL of hydrochloric acid was added and mixed well. The sample was placed in a 75°C water bath for hydrolysis for 40 min, the flask was shaken every 10 min, and after hydrolysis, it was cooled to room temperature.

A total of 10 mL of 95% ethanol was added to the hydrolyzed sample and mixed well. The hydrolysate in the flask was transferred to a separating funnel; the flask and plug were washed with 50 mL of mixed solution of ether and petroleum ether, and the washing solution was added to the separating funnel and covered. The mixture was shaken for 5 min and allowed to stand for 10 min. The ether layer extract was collected into a 250 mL flask. The above steps were repeated to extract the hydrolysate three times. Finally, the separating funnel was washed with a mixture of ether and petroleum ether and collected into a constant weight flask. The flask was steamed in a water bath and dried in an oven at 100°C ± 5°C for 2 h.

In the organic solvent extract, 2 mL of 2% sodium hydroxide methanol solution was added to the 85°C water bath for 30 min, and 3 mL of 14% boron trifluoride methanol solution was added to the 85°C water bath for 30 min. After the water bath was completed, the solution was allowed to cool to room temperature; 1 mL n‐hexane was added to the centrifuge tube, shaken, and extracted for 2 min; and the mixture was then allowed to stand for 1 h for stratification. A total of 100 μL supernatant was collected. The supernatant was diluted to 1 mL with n‐hexane and filtered through a 0.25 μm polyester filter for analysis.

The GC conditions were as follows: the model of the column was TG‐5MS GC (30 m × 0.25 mm × 0.25 μm film thickness). The initial column temperature was 80°C and kept for 1 min. Then, the temperature was raised to 200°C at 10°C/min, then to 250°C at 5°C/min, held for 5 min, raised to 270°C at 2°C/min, and held for 8 min. The carrier gas was high‐purity He (99.999%), and the gas flow rate was 1.2 mL/min with splitless injection mode. The injector temperature was 290°C. The MS conditions were set as follows: the EI ionization source temperature was set to 280°C. The electron energy was set at 70 eV, and the mass range was between 30 and 400 amu.

### 2.7 Odor Change by E‐Nose

The odor change was determined according to Li et al. (2019) with a PEN 3 Portable Electronic Nose (E‐nose) (Airsense Analytics Inc., Schwerin, Germany). The E‐nose system included 10 metal oxide semiconductor sensors: W1C (aromatic compounds), W5S (oxynitride), W3C (ammonium hydroxide and aroma component), W6S (hydrocarbons), W5C (alkanes and aromatics), W1S (methane), W1W (sulfur compounds), W2S (alcohols), W2W (aromatics and organic sulfur compounds), and W3S (alkane). A total of 3 g of chicken meat was placed in a 20 mL headspace bottle, sealed, and equilibrated for 10 min. The measurement time and flushing time were 120 and 5 s, respectively. Three duplicate samples were prepared for each experiment. The response values of the E‐nose were recorded and analyzed by principal component analysis (PCA), linear discriminant analysis (LDA), and loading analysis (LA).

### 2.8 Volatile Compound Determination

Volatile compounds of chicken meat were isolated by adopting HS‐SPME and were measured according to Xie et al. (2018) with some modifications. Solid‐phase silica fibers with 100 μm polydimethylsiloxane coating and a manual SPME holder were purchased from Supelco (Bellefonte, PA, USA). Before use, fibers were conditioned for 5 min according to the manufacturer's recommended conditioning temperature.

A total of 3.0 g of chicken meat was accurately weighed for each group and placed in a 20 mL headspace vial sealed with a cap. The headspace vials were maintained at 65°C for 30 min. Then, fibers were exposed to the headspace of the sample for 30 min of extraction to ensure that volatile compounds were absorbed by SPME fibers. Fibers were pulled out from the headspace bottle immediately after extraction and inserted into the GC injector for desorption (250°C for 15 min) and analysis.

A GC–MS instrument (GC: Trace 1310; MS: ISQ, ThermoFisher, USA) and HP‐5MS GC column were used (60 m × 0.25 mm × 0.25 μm film thickness) to determine volatile compounds, and high‐purity helium (> 99.999%) served as the carrier gas at a flow rate of 1.6 mL/min with splitless injection mode. The initial oven temperature was set at 50°C for 5 min, increased at 3°C/min to 120°C, held for 3 min, increased at 4°C/min to 180°C, held for 3 min, increased to 250°C at 5°C/min, and held for 20 min. The injector temperature was 250°C. The MS parameters were as follows: the ion source temperature was adjusted to 320°C, with data obtained in full scan mode. The volatile compounds were identified using the National Institute of Standards and Technology (NIST) library.

### 2.9 Statistical Analysis

The significant differences among the groups were analyzed by SPSS version 22.0 software (SPSS Inc., Chicago, IL). To test for the treatment effect, the data were analyzed by one‐way analysis of variance (ANOVA). Mean separations were performed by Duncan's multiple‐range tests. Differences at *p* < 0.05 were considered statistically significant. Data are expressed as the means ± standard errors (SEs).

## 3 Results and Discussion

### 3.1 Dosimetry

The actual absorbed dose was measured by a silver dichromate dosimeter. The dose eventually absorbed by chicken meat was 4.22 kGy.

### 3.2 Microbiological Assessment

To estimate the effect of 4 kGy gamma irradiation combined with antioxidants on the microbial quality of spicy chicken meat, five kinds of microbes (total viable bacteria, total coliform counts, *Salmonella* spp., *Staphylococcus aureus* , *Listeria monocytogenes* ) that clearly limit demands in the provincial standard of Guizhou DBS52001‐2014 were tested. The results are shown in Table 1. *Salmonella* spp., *S. aureus* , and *L. monocytogenes* were not detected in the nonirradiated and irradiated samples. As the storage period increased, an elevated count of total viable bacteria (TVB) was observed in the 0 kGy and 0 kGy + A groups during the whole storage time. The TVB after irradiation with 4 kGy and 4 kGy + A gradually decreased until the thirtieth day and then increased in the rest of the storage period. The TVB in all irradiated groups significantly (*p* < 0.05) decreased with increasing storage time compared with that in the 0 kGy and 0 kGy + A groups. There was no significant difference between the 4 kGy and 4 kGy + A groups. The same results were found in the 0 kGy and 0 kGy + A groups. The total coliform counts (TCCs) in all group samples were lower than 3.0 MPN/g in the first 30 days. On the 45th and 60th days, the TCCs in the 0 kGy and 0 kGy + A groups were dramatically increased at the end of storage to 8.75 and 8.71 MPN/g TCCs in 0 kGy and 0 kGy + A, respectively; however, the TCCs in the 4 kGy and 4 kGy + A groups were still below 3.0 MPN/g. The results indicated that meat irradiated with 4 kGy could efficiently inhibit the reproduction of TVB and TCC, and that 4 kinds of antioxidants had no direct antibacterial effect on TVB and TCC in chicken meat. *Salmonella* spp., *S. aureus* , and *L. monocytogenes* were not detected in all treatment groups, mainly because the chicken meat was boiled and fried at high temperatures to eliminate those three pathogenic bacteria.

**TABLE 1:** Irradiation combined with antioxidants on the microbiological qualities of spicy chicken meat.

Time (d)	Treatments	Total viable bacteria (log CFU × g^−1^)	Total coliform counts (MPN × g^−1^)	*Salmonella* spp (CFU × g^−1^)	*Staphylococcus aureus* (CFU × g^−1^)	*Listeria monocytogenes* (CFU × g^−1^)
0	0 kGy	4.21 ± 0.03a	< 3.0	ND	ND	ND
	0 kGy + A	4.21 ± 0.02a	< 3.0	ND	ND	ND
	4 kGy	3.21 ± 0.09b	< 3.0	ND	ND	ND
	4 kGy + A	3.15 ± 0.07b	< 3.0	ND	ND	ND
15	0	4.38 ± 0.02a	< 3.0	ND	ND	ND
	0 kGy + A	4.36 ± 0.02a	< 3.0	ND	ND	ND
	4 kGy	2.61 ± 0.12b	< 3.0	ND	ND	ND
	4 kGy + A	2.71 ± 0.06b	< 3.0	ND	ND	ND
30	0	4.48 ± 0.04a	< 3.0	ND	ND	ND
	0 kGy + A	4.44 ± 0.02a	< 3.0	ND	ND	ND
	4 kGy	2.30 ± 0.01b	< 3.0	ND	ND	ND
	4 kGy + A	2.37 ± 0.11b	< 3.0	ND	ND	ND
45	0	4.83 ± 0.03a	5.73 ± 1.19	ND	ND	ND
	0 kGy + A	4.81 ± 0.03a	5.66 ± 0.28	ND	ND	ND
	4 kGy	2.32 ± 0.15b	< 3.0	ND	ND	ND
	4 kGy + A	2.26 ± 0.07b	< 3.0	ND	ND	ND
60	0	5.06 ± 0.05a	8.75 ± 1.33	ND	ND	ND
	0 kGy + A	5.02 ± 0.06a	8.71 ± 0.31	ND	ND	ND
	4 kGy	2.57 ± 0.04b	< 3.0	ND	ND	ND
	4 kGy + A	2.49 ± 0.06b	< 3.0	ND	ND	ND

*Note:* Data represent the mean values ± standard deviations (*n* = 3). Values with different letters at each time point are significantly different according to Duncan's multiple range test (*P* < 0.05).

Abbreviation: ND, not detected.

Irradiation is one of the safest methods to control the quality and safety of meat and meat products (Islam et al. 2019). Gamma irradiation has been applied in the meat processing industry for disinfection for decades and can be used not only to maintain safety but also to improve the quality of meat (Hassanzadeh et al. 2017; Artes et al. 2007). Chicken meat may be contaminated by many species of microorganisms during processing, transportation, and handling (Arshad et al. 2019). Irradiation can reduce the contamination level of microbes depending on the energy source, dose rate, and absorbed dose (Ahn and Lee 2006; Kyung et al. 2019). Wellington et al. (2014) reported that irradiation doses of 4 to 8 kGy in combination with frozen storage could effectively reduce TVB and *E. coli* . Even a low dose of gamma also stops the reproduction of Enterococci spp. and *Escherichia coli* . The mechanism of sterilization of irradiation was clarified by previous researchers, who showed that the DNA of microorganisms was destroyed by direct irradiation or indirect effects through the production of radicals and ions, breaking phosphodiester bonds in the DNA and resulting in the loss of a cell's ability to replicate (Jayathilakan and Sultana 2018).

### 3.3 Hunter's Color

The color on the surface of spicy chicken is mainly determined by the chili oil, which contains abundant capsanthin (Konishi et al. 2019). Irradiation significantly (*p* < 0.05) increased the ΔL* values over the whole storage period (Table 2). Moreover, the antioxidants significantly (*p* < 0.05) inhibited ΔL* growth at 0, 30, 45, and 60 days. The value of ΔL* in the 4 kGy group decreased in the first 15 days and then gradually increased. On the 60th day, the ΔL* value of the 4 kGy group was −9.30, and 4 kGy + A was 54.95% lower than that of the 4 kGy group. The results showed that the Δa* value was significantly (*p* < 0.05) increased in the 4 kGy + A group compared with the 4 kGy group over the whole storage time. The Δa* value in the 4 kGy and 4 kGy + A groups ranged from −12.80 to −9.30 and −15.85 to 14.41, respectively. The Δa* value in the 4 kGy + A group decreased gradually during the first 15 days and remained steady during the rest of the storage time. The 4 kGy irradiation combined with antioxidant treatment significantly (*p* < 0.05) enhanced the Δb* value at 30, 45, and 60 days after treatment, with values 10.56%, 17.32%, and 47.25% higher, respectively, than those in the 4 kGy group at these time points. The Δb* value in the 4 kGy group sharply decreased with storage time; however, the Δb* value in the 4 kGy + A group peaked on the 30th day, and then the value slightly increased. A similar variation trend was observed for ΔE* values of the 4 kGy and 4 kGy + A groups. The 4 kGy + A treatment significantly (*p* < 0.05) inhibited the reduction in the ΔE* value at 45 and 60 days.

**TABLE 2:** 4 kGy gamma‐ray combined with antioxidants on Hunter's color values of spicy chicken during storage.

Storage time (d)	Treatments	ΔL*	Δa*	Δb*	ΔE*
0	0 kGy	−17.58 ± 0.92a	17.54 ± 0.62a	45.57 ± 3.11ab	57.90 ± 3.16ab
	0 kGy + A	−18.41 ± 0.65a	17.33 ± 0.24a	47.19 ± 2.45a	53.53 ± 2.42a
	4 kGy	−12.8 ± 0.66c	14.13 ± 0.65b	38.03 ± 2.13c	42.54 ± 2.60c
	4 kGy + A	−15.85 ± 1.06b	16.81 ± 1.05a	42.14 ± 1.23bc	48.06 ± 2.51b
15	0	−18.08 ± 1.10a	15.49 ± 1.05ab	46.64 ± 2.05a	52.37 ± 3.23a
	0 kGy + A	−17.87 ± 0.39a	16.21 ± 0.71a	46.52 ± 3.36a	52.41 ± 3.37a
	4 kGy	−13.96 ± 1.12b	12.6 ± 0.80c	37.44 ± 2.33b	41.90 ± 3.79b
	4 kGy + A	−15.11 ± 0.53b	14.56 ± 0.69b	41.62 ± 1.85b	46.61 ± 1.89ab
30	0	−17.82 ± 1.25a	16.58 ± 0.92a	44.07 ± 1.66a	50.34 ± 2.79a
	0 kGy + A	−18.22 ± 0.46a	15.87 ± 0.14a	43.28 ± 0.81a	49.57 ± 0.51a
	4 kGy	−12.46 ± 0.53b	11.07 ± 0.33c	33.29 ± 0.85c	37.22 ± 1.00b
	4 kGy + A	−14.45 ± 1.72b	14.23 ± 0.75b	36.80 ± 1.35b	42.77 ± 2.37b
45	0	−16.59 ± 0.30a	16.30 ± 0.04a	45.56 ± 0.42a	51.15 ± 0.32a
	0 kGy + A	−16.53 ± 1.17a	15.93 ± 0.22a	44.64 ± 1.60a	50.19 ± 0.49a
	4 kGy	−10.95 ± 0.67c	10.63 ± 0.18c	31.99 ± 1.64c	35.44 ± 1.27c
	4 kGy + A	−14.5 ± 0.93b	14.51 ± 0.40b	37.53 ± 2.47b	42.77 ± 2.38b
60	0	−16.95 ± 0.47a	16.46 ± 0.40a	43.17 ± 1.18a	49.21 ± 1.32a
	0 kGy + A	−16.06 ± 0.53a	15.52 ± 0.63a	45.02 ± 1.47a	50.25 ± 1.74a
	4 kGy	−9.30 ± 0.44c	9.88 ± 0.79c	27.16 ± 1.79c	30.36 ± 1.83c
	4 kGy + A	−14.41 ± 0.55b	14.45 ± 0.39b	39.99 ± 1.43b	44.90 ± 1.07b

*Note:* Values with different letters at each time point are significantly different according to Duncan's multiple range test (*P* < 0.05).

The values of ΔL*, Δa*, and Δb* represent lightness, redness, and yellowness, respectively. The apparent color of spicy chicken is mainly determined by the chili oil, which contains abundant capsanthin. Capsanthin is a type of red carotenoid pigment that is synthesized during ripening and is responsible for the final red color in chili (Kyung et al. 2019). Our results showed that lightness, redness, and yellowness were sensitive to gamma irradiation at a dose of 4 kGy. Similar results were found in a previous study; the yellow (𝛽‐carotene, 𝛽‐cryptoxanthin, zeaxanthin and capsolutein) and red (capsanthin) pigments of the carotenoid family are very sensitive to irradiation (Kyung et al. 2019). The capsanthin fading caused by irradiation mainly occurs because of direct oxidation or addition reactions in the presence of reactive oxygen species (ROS), which are produced by irradiation (Ding et al. 2015). The autoxidation process of unsaturated fatty acids of the oil in spicy chicken samples was accelerated by irradiation and produced various oxygen‐containing products, such as hydroperoxides and carbonyl compounds, which reacted with capsanthin, resulting in fading (Jung et al. 2015). The antioxidants could react with strong oxidation substances (ROS, hydroperoxides, and carbonyl compounds) before capsanthin to prevent color fading. Antioxidants have been demonstrated to improve color stability in irradiated chicken meat (Yim et al. 2015; Nam and Ahn 2003). Vitamin E and α‐tocopherol have the ability to scavenge free radicals and stop progressive autoxidative damage in meat (Morrissey et al. 1998; Yim et al. 2015).

### 3.4 POV

**FIGURE 1:**
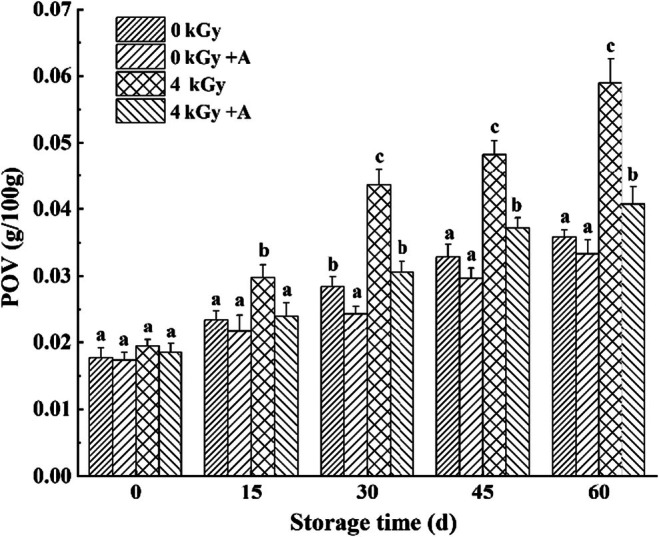
Effect of 4 kGy gamma‐ray irradiation with antioxidants on peroxide values of spicy chicken during storage. Each column represents the mean of three replicates. Bars represent standard deviations of the means. Columns with different letters at each time point are significantly different according to Duncan's multiple range test (*P* < 0.05).

Peroxide values (POVs) indicate the level of oxidative degradation of lipids from spicy chicken. The POV in the 4 kGy irradiated group dramatically and significantly increased after 15 days of storage in comparison with other treatments. On the other hand, the 4 kGy + A treatment obviously inhibited an increase in POVs, with values that were 19.38%, 29.86%, 22.93%, and 30.97% significantly lower (*p* < 0.05) than those in the 4 kGy treatment (Figure 1). Our results showed that the POV decreased in irradiated chicken samples combined with four kinds of antioxidants compared to irradiated samples without antioxidant treatment.

Lipid oxidation can be divided into different stages according to the sequence of chemical changes taking place. Peroxides were reported as the first products of lipid oxidation, and POV was used to indicate oxidation in early stages (Mehta et al. 2015; Zheng et al. 2022). Many studies have demonstrated that several substances (turmeric powder, leek extract, grape seed extract, and cassia essential oil) with high antioxidant activity can suppress lipid oxidation and inhibit the increase in POV in meat (Arshad et al. 2019; Kim et al. 2013; Hassanzadeh et al. 2017). Long et al. (2019) showed that phytic acid exhibited potent hydroxyl radical scavenging activity, while tea polyphenols had powerful 1,1‐diphenyl‐2‐picrylhydrazyl (DPPH) radical scavenging activity, and the best synergistic antioxidant effect in irradiated chicken wings was observed with a 1:1 mixture of phytic acid and tea polyphenols. Our results showed that the combination of four kinds of antioxidants, α‐tocopherol, phytic acid, tea polyphenols, and TBHQ, also demonstrated powerful antioxidant capacity, suppressing the increase in POV caused by irradiation.

### 3.5 Fatty Acids

Table 3 shows the fatty acid compositions of spicy chicken samples treated with 4 kGy irradiation without antioxidants and with antioxidants. The major components of fatty acids (≥ 5.00 mg/100 g) found in both irradiated and nonirradiated samples were palmitic acid (C16:0), oleic acid (C18:1n9c), linoleic acid (C18:2n6c), and α‐linolenic acid (C18:3n3), with values that ranged from 7.26 to 7.65, 31.91 to 37.28, 27.93 to 34.12, and 5.7 to 7.89 mg/100 g, respectively (Table 2). The results demonstrated that saturated fatty acids (SFAs), monounsaturated fatty acids (MUFAs), and polyunsaturated fatty acids (PUFAs) showed significant (*p* ≤ 0.05) changes under different treatments.

**TABLE 3:** Irradiation combined with antioxidants on fatty acids of spicy chicken meat at the beginning of storage (0 day) and the end of storage (60 days).

Fatty acids (mg/100 g)	Storage days (d)							
	0				60			
**SFAs**	0 kGy	0 kGy + A	4 kGy	4 kGy + A	0 kGy	0 kGy + A	4 kGy	4 kGy + A
C14:0	0.08 ± 0.02a	0.07 ± 0.01a	0.08 ± 0.03a	0.09 ± 0.01a	0.21 ± 0.03a	0.22 ± 0.01a	0.19 ± 0.02a	0.21 ± 0.02a
C16:0	7.48 ± 0.06a	7.27 ± 0.09b	7.55 ± 0.12c	7.49 ± 0.03d	7.26 ± 0.03a	7.57 ± 0.06b	7.43 ± 0.02c	7.65 ± 0.03d
C17:0	ND	ND	ND	ND	0.14 ± 0.01a	0.14 ± 0.02a	0.13 ± 0.02a	0.14 ± 0.02a
C18:0	3.63 ± 0.02 a	3.61 ± 0.04a	3.47 ± 0.07b	3.59 ± 0.08a	3.96 ± 0.05a	4.03 ± 0.10a	3.58 ± 0.14b	3.61 ± 0.04b
C20:0	0.29 ± 0.01a	0.29 ± 0.03a	0.31 ± 0.01a	0.30 ± 0.02a	0.41 ± 0.02a	0.37 ± 0.03a	0.66 ± 0.03b	0.42 ± 0.02a
C22:0	0.16 ± 0.01a	0.17 ± 0.01a	0.17 ± 0.02a	0.17 ± 0.01a	0.22 ± 0.01a	0.22 ± 0.03a	0.27 ± 0.03b	0.22 ± 0.03a
C23:0	ND	ND	ND	ND	0.07 ± 0.02a	0.07 ± 0.01a	0.08 ± 0.02a	0.07 ± 0.01a
C24:0	0.10 ± 0.02a	0.09 ± 0.01a	0.11 ± 0.01a	0.12 ± 0.03a	0.14 ± 0.03a	0.13 ± 0.02a	0.17 ± 0.02a	0.14 ± 0.02a
Total	11.74 ± 0.07a	11.50 ± 0.11b	11.69 ± 0.15ab	11.77 ± 0.11a	12.41 ± 0.07a	12.75 ± 0.12b	12.51 ± 0.18a	12.46 ± 0.06a
**MUFAs**								
C16:1n7	0.41 ± 0.02a	0.42 ± 0.01a	0.34 ± 0.03b	0.38 ± 0.02a	0.43 ± 0.01ab	0.41 ± 0.02b	0.30 ± 0.02c	0.46 ± 0.02a
C18:1n9c	37.28 ± 0.38a	37.21 ± 0.24a	35.03 ± 0.18b	36.37 ± 0.11c	34.22 ± 0.22a	35.46 ± 0.13b	31.91 ± 0.16c	33.21 ± 0.11d
C20:1	0.63 ± 0.04a	0.65 ± 0.03a	0.45 ± 0.02b	0.47 ± 0.03b	0.59 ± 0.03a	0.62 ± 0.03a	0.43 ± 0.02b	0.58 ± 0.03a
C22:1n9	0.16 ± 0.03a	0.17 ± 0.01a	0.10 ± 0.02b	0.18 ± 0.03a	0.16 ± 0.02a	0.16 ± 0.02a	0.09 ± 0.02b	0.14 ± 0.02a
C24:1n9	0.13 ± 0.01a	0.13 ± 0.01a	0.05 ± 0.03b	0.07 ± 0.01b	0.13 ± 0.01a	0.12 ± 0.01a	ND	0.08 ± 0.02b
Total	38.62 ± 0.41a	38.58 ± 0.28a	35.96 ± 0.22b	37.47 ± 0.15c	35.53 ± 0.27a	36.77 ± 0.16b	32.73 ± 0.19c	34.47 ± 0.15d
**PUFAs**								
C18:2n6c	34.12 ± 0.15a	34.69 ± 0.06b	31.33 ± 0.23c	33.68 ± 0.12b	32.72 ± 0.22a	32.31 ± 0.14b	27.93 ± 0.16c	30.33 ± 0.19d
C18:3n6	0.23 ± 0.03a	0.24 ± 0.02a	0.12 ± 0.04c	0.20 ± 0.02b	ND	ND	ND	ND
C18:3n3	7.89 ± 0.11a	7.85 ± 0.04a	6.21 ± 0.03b	6.73 ± 0.06c	7.32 ± 0.05a	7.27 ± 0.06a	5.70 ± 0.08c	7.03 ± 0.1b
C20:2	0.23 ± 0.03a	0.22 ± 0.01a	0.21 ± 0.02a	0.30 ± 0.02b	0.13 ± 0.01ab	0.11 ± 0.02b	0.17 ± 0.03a	0.14 ± 0.03ab
C20:3n6	0.13 ± 0.02a	0.13 ± 0.01a	0.09 ± 0.02a	0.10 ± 0.03a	0.07 ± 0.02a	0.05 ± 0.01a	0.04 ± 0.01a	0.05 ± 0.02a
C20:4n6	1.14 ± 0.03a	1.11 ± 0.05a	1.09 ± 0.04a	1.86 ± 0.06b	1.16 ± 0.06a	1.15 ± 0.03a	0.91 ± 0.04b	1.09 ± 0.05a
C20:5n3	0.16 ± 0.04a	0.16 ± 0.02a	0.14 ± 0.02a	0.14 ± 0.03a	0.14 ± 0.03a	0.12 ± 0.01a	0.06 ± 0.02b	0.07 ± 0.02b
C22:6n3	0.25 ± 0.03ab	0.27 ± 0.02ab	0.30 ± 0.02a	0.27 ± 0.02ab	0.27 ± 0.03a	0.26 ± 0.02ab	0.20 ± 0.02b	0.26 ± 0.05ab
Total	44.15 ± 0.16a	44.67 ± 0.11b	39.49 ± 0.17c	43.27 ± 0.13d	41.81 ± 0.25a	41.27 ± 0.18b	35.01 ± 0.19c	38.97 ± 0.24d

*Note:* Data represent the mean values ± standard deviations (n=3).Values with different letters at each time point are significantly different according to Duncan’s multiple range test (*p* < 0.05).

Abbreviation: ND, not detected.

The content of total SFAs in spicy chicken samples without the addition of antioxidants was 11.74 mg/100 g; after 4 kGy γ‐irradiation, the content decreased by 0.43%, while the SFA contents in spicy chicken samples with 0 kGy irradiation and the addition of antioxidants showed a slight increase from 11.50 to 11.77 mg/100 g. However, after storage for 60 days, the SFA content was 12.41 mg/100 g with 0 kGy treatment and increased by 0.81% to a value of 12.51 mg/100 g with 4 kGy treatment. In the 0 kGy + A and 4 kGy + A groups, the values were 12.75 and 12.46 mg/100 g, respectively, representing a decrease of 2.27%. The contents of total MUFAs in the 0 kGy and 0 kGy + A groups were 38.62 and 38.58 mg/100 g, respectively. Exposure to irradiation at 4 kGy resulted in decreases of 6.89% and 2.88%, respectively. At the end of storage, the MUFA values of the four groups were all reduced compared with those at the beginning of storage, with values of 35.53, 36.77, 32.73, and 34.47 mg/100 g, respectively. The total PUFA contents in the 0 kGy and 0 kGy + A groups were 44.15 and 44.67 mg/100 g, respectively, and significantly (*p* ≤ 0.05) decreased in the 4 kGy and 4 kGy + A groups, with values that were 10.65% and 3.13% lower than those in the 0 kGy and 0 kGy + A groups, respectively. After storage for 60 days, the PUFA contents were 41.81 and 41.27 mg/100 g in the 0 kGy and 0 kGy + A groups, respectively, and exhibited decreases of 16.26% and 5.57% when they were exposed to irradiation at 4 kGy. Trans fatty acids were not observed in irradiated and nonirradiated chicken meat.

The fatty acid content of meat affects its quality characteristics, such as flavor, texture, and aroma. The factors affecting the content of fatty acids in meat include the species of native animals, processing methods, and preservation methods (Jo et al. 2018). Chicken meat contains relatively high amounts of unsaturated fatty acids that make chicken meat considerably susceptible to deterioration caused by oxidation processes (Xiao et al. 2011). Our results are in accordance with Jo et al. (2018), who observed that irradiation at 4 kGy decreased the content of unsaturated fatty acids in duck meat. Mahrour et al. (2003) also found that the concentration of UFAs decreased and the concentration of SFAs in the meat of chicken legs increased after irradiation with 5 kGy; moreover, the levels of UFAs in chicken legs irradiated with natural antioxidants were higher than those irradiated without antioxidants. The free radicals (OH^·−^, hydrated electron, and H^+^) produced by the radiolysis of water (Jayathilakan and Sultana 2018). A free radical would abstract a hydrogen from a fatty acid, and the fatty acid becomes a lipid radical, which may attack the double bond position (Huang and Ahn 2019). Therefore, PUFAs are more susceptible to radiolysis than monounsaturated or saturated fatty acids, and irradiation causes a significant reduction in PUFAs. Antioxidants are much more competitive for reactions with free radicals than PUFAs (Xiao et al. 2011). The incorporation of antioxidants with free radical scavenging activities helped protect against lipid peroxidation in irradiated meat and meat products (Jayathilakan and Sultana 2018).

### 3.6 E‐Nose

#### 3.6.1 Principal Component Analysis

**FIGURE 2:**
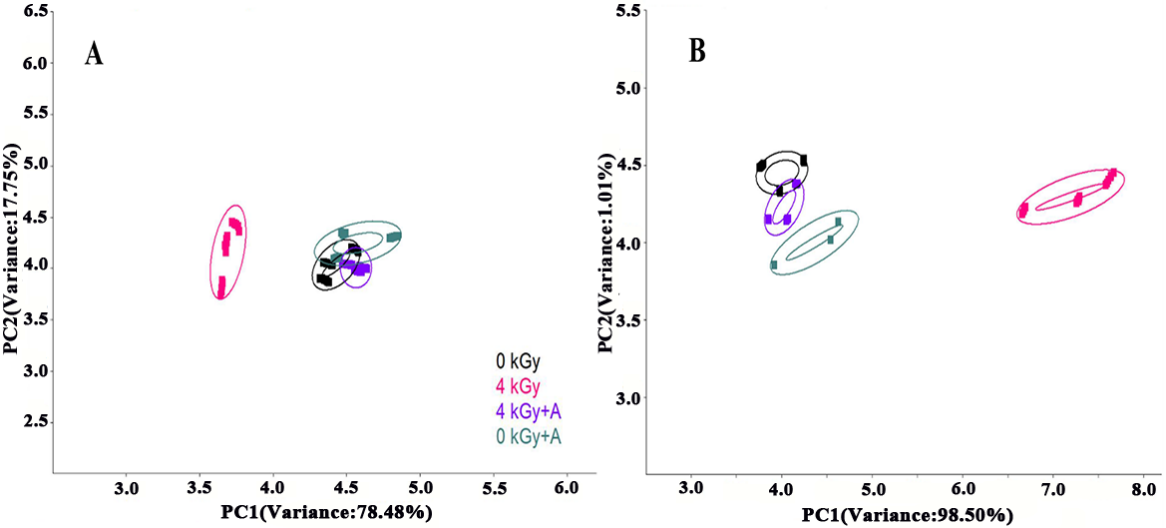
PCA of spicy chicken meat irradiated with a dose of 4 kGy at 0 days (A) and 60 days (B). 0 kGy + A and 4 kGy + A indicate irradiation with four kinds of antioxidants and without four kinds of antioxidants, respectively.

**FIGURE 3:**
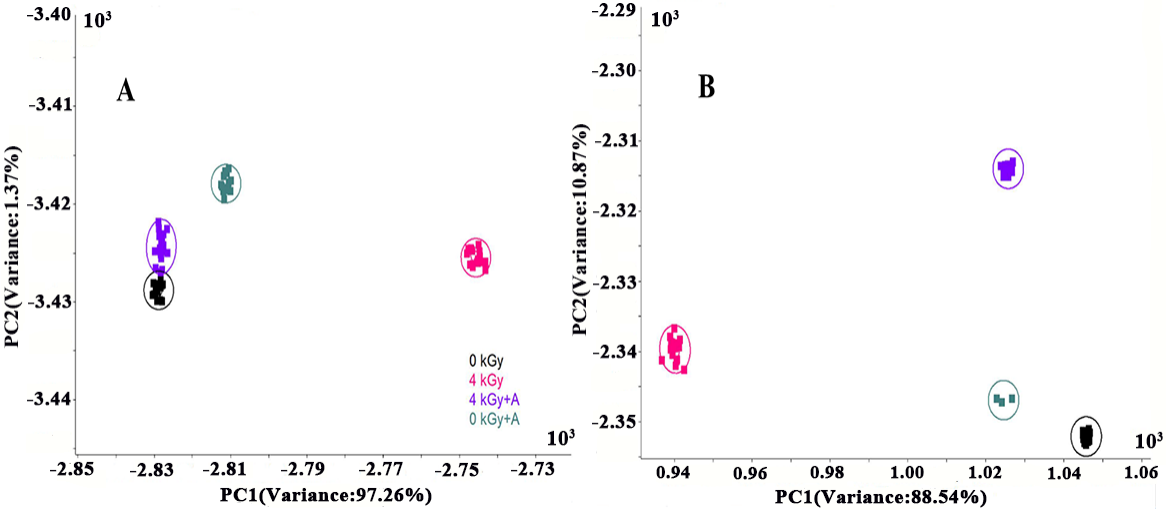
LDA of spicy chicken meat irradiated with a dose of 4 kGy at 0 days (A) and 60 days (B). 0 kGy + A and 4 kGy + A indicate irradiation with four kinds of antioxidants and without four kinds of antioxidants, respectively.

Principal component analysis (PCA) of spicy chicken samples was performed beginning (0 days) and ending (60 days) of storage (Figure 2). PCA was presented to illustrate the odor of spicy chicken. The first principal component (PC1) and the second principal component (PC2) accounted for 78.48% and 17.75% of the total variations, respectively, at 0 days of storage (Figure 3A). For comparison, PC1 explained 98.50% of the total variance with a value of 99.51, whereas PC2 accounted for 1.01% at the end of storage (Figure 3B). The accumulative variance rates of these two major components were larger than 80%, which meant that PC1 and PC2 present the majority of odor information (Huang et al. 2019). At the beginning of storage, the cluster of the 4 kGy + A group overlapped with the clusters of the 0 kGy and 0 kGy + A groups, and the cluster of the 4 kGy group was far away from those three clusters along PC1, which indicated that irradiation with or without antioxidants had different effects on the odor of spicy chicken. Irradiation with antioxidants has a slight effect on odor; in contrast, the effect of irradiation without antioxidants on flavor was more apparent. Similarly, during 60 days of storage, the distribution areas of the 0 kGy and 4 kGy + A groups still overlapped, and the odor of the 0 kGy + A group was close to that of the 4 kGy + A group. These three clusters were not distinguished according to PC1. The distribution area of the 4 kGy group was far from the other three sample groups.

#### 3.6.2 Linear Discriminant Analysis

**FIGURE 4:**
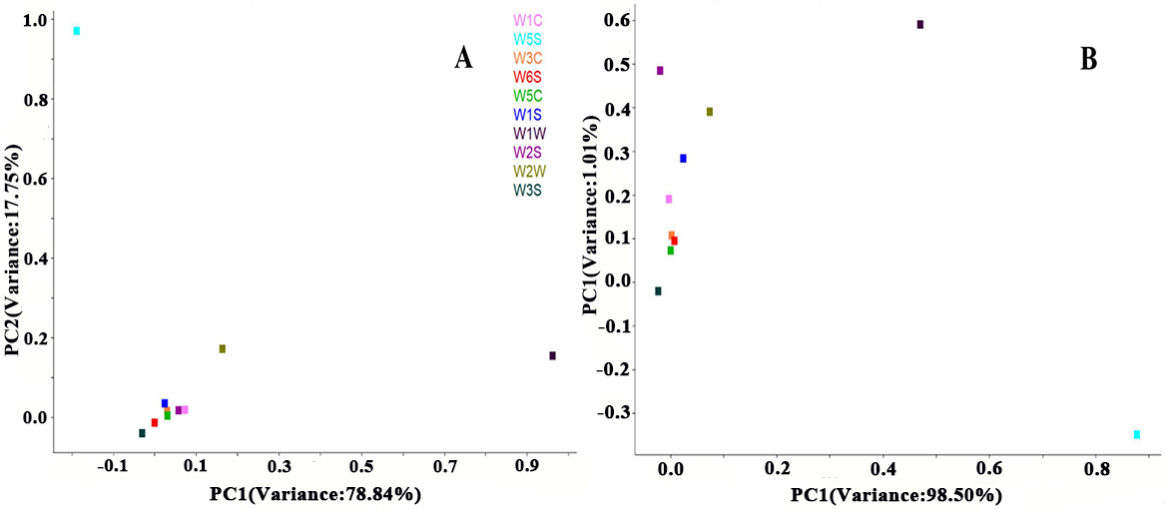
LA plots of spicy chicken meat irradiated with a dose of 4 kGy at 0 days (A) and 60 days (B).

Linear discriminant analysis (LDA) was used to find a linear transformation to achieve maximum class discrimination (Zhou 2017). Figure 4 shows the LDA of spicy chicken samples at storage for 0 days and 60 days. The contribution rates of PC1 and PC2 were 97.26% and 1.37%, respectively. The clusters of 0 kGy and 4 kGy samples overlapped slightly, and 0 kGy + A samples were near 0 kGy and 4 kGy samples along both PC1 and PC2. The 4 kGy group was completely separated from the other three groups with long distances (Figure 3A). At the end of storage, clusters of the 4 kGy + A and 0 kGy + A groups were not distinguished and were close to the 0 kGy group along PC1. The 4 kGy group still showed a longer distance from the 0 kGy group than the 4 kGy + A and 0 kGy + A groups along PC1 (Figure 3B).

#### 3.6.3 Loading Analysis

Loading analysis (LA) usually evaluates the contribution of ten sensors responsible for distinguishing the current pattern. One plot presents a metal oxide semiconductor sensor; a plot near zero indicates a low contribution in the loading pattern, whereas a long distance from zero indicates a high contribution. Sensor W1W plays an important role on PC1, followed by W2W. For PC2, W5S exhibited a remarkable influence, followed by W2W (Figure 4A). However, after 60 days of storage, sensor W5S became a key sensor that showed a significant influence on PC1, and W1W became the second most important sensor, followed by W5S. W1W also significantly influenced PC2, followed by W2S, W2W, and W1S. Other sensors were near the zero point, which indicated that their influence was negligible (Figure 4B).

### 3.7 Volatile Compounds

A total of 31 kinds of volatile compounds were identified in spicy chicken, including acids, alcohols, alkenes, esters, aldehydes, ketones, aromatic hydrocarbons, furans, sulfur compounds, and others (Table 4). Alkenes are the most volatile components in spicy chicken, followed by esters and alcohols. D‐Limonene, linalyl acetate, and linalool were the major components in alkenes, esters, and alcohols, respectively. Irradiation and antioxidant addition had different effects on the volatile compounds of spicy chicken. Acetic acid was detected in chicken samples without antioxidants but was detected in samples with antioxidants at 0 days of storage. After irradiation, 1,3‐bis(1,1‐dimethylethyl)‐benzene was detected in the samples after both 0 days and 60 days of storage. The alkene contents decreased after 4 kGy irradiation, but after 60 days of storage, the alkene contents increased to varying degrees. In the 4 kGy + A group, the alkene content was the highest, with a value of 52.83 ± 1.80. A previous study confirmed that sulfur‐containing compounds could be the major volatile components responsible for the characteristic off‐odor in irradiated meat (Ahn et al. 2000). Because volatile flavor profiles consist of diverse classes of compounds, the effect of irradiation treatment may vary depending on the specific and individual class of volatile compounds (Gyawali et al. 2008). Lipid oxidation and the radiolytic breakdown of sulfur‐containing amino acids are important factors affecting volatile compounds in foods (Ahn and Lee 2006). In our study, no new sulfur‐containing compounds were produced and only slightly increased in the 4 kGy group; however, at the end of storage, the content of sulfur‐containing compounds decreased from 4.52% ± 0.10% to 1.58% ± 0.11% in the 4 kGy group and from 5.67% ± 0.17% to 1.85% ± 0.06% in the 4 kGy + A group. Nam and Ahn (2003) mentioned that vacuum conditions and antioxidants could minimize the generation of volatilizing sulfur‐containing compounds and restrain lipid oxidation in pork meat.

**TABLE 4:** The types and relative contents of volatile compounds in irradiated spicy chicken at the beginning of storage (0 day) and the end of storage (60 days).

Volatile compounds	0 day				60 days			
	0 kGy	0 kGy + A	4 kGy	4 kGy + A	0 kGy	0 kGy + A	4 kGy	4 kGy + A
**Acids**								
Acetic acid	ND	2.94 ± 0.24	ND	4.83 ± 0.47	ND	ND	ND	ND
**Alcohols**								
Ethanol	4.54 ± 0.12	2.92 ± 0.11	3.66 ± 0.43	1.56 ± 0.14	1.57 ± 0.47	1.59 ± 0.07	0.72 ± 0.03	0.66 ± 0.02
Linalool	10.12 ± 0.42	8.28 ± 0.63	12.89 ± 0.39	10.41 ± 0.11	12.64 ± 0.41	14.95 ± 1.33	11.12 ± 0.57	14.15 ± 1.09
Terpinen‐4‐ol	2.83 ± 0.11	2.45 ± 0.19	3.44 ± 0.07	2.73 ± 0.02	3.29 ± 0.05	3.23 ± 0.04	2.38 ± 0.21	2.30 ± 0.17
α‐Terpineol	0.73 ± 0.05	0.52 ± 0.04	0.88 ± 0.02	0.75 ± 0.05	0.53 ± 0.02	0.51 ± 0.04	0.68 ± 0.03	0.43 ± 0.04
2,6‐Octadien‐1‐ol, 3,7‐dimethyl‐, acetate	0.60 ± 0.03	0.59 ± 0.04	0.55 ± 0.02	0.55 ± 0.01	ND	ND	ND	ND
2‐Furanmethanol	0.87 ± 0.06	0.27 ± 0.03	1.10 ± 0.02	0.65 ± 0.04	ND	ND	ND	ND
subtotal	20.22 ± 0.48	15.02 ± 0.50	22.52 ± 0.08	16.65 ± 0.09	18.02 ± 0.10	20.28 ± 1.26	14.65 ± 0.84	17.53 ± 1.27
**Alkenes**								
ß‐Myrcene	7.24 ± 0.25	6.83 ± 0.35	6.30 ± 0.28	5.00 ± 0.18	9.14 ± 0.29	9.16 ± 0.07	8.23 ± 0.81	9.72 ± 1.17
α‐Phellandrene	0.43 ± 0.06	0.39 ± 0.02	0.47 ± 0.03	0.25 ± 0.01	0.35 ± 0.03	0.41 ± 0.03	0.44 ± 0.03	0.38 ± 0.05
α‐Terpinene	2.14 ± 0.13	1.79 ± 0.26	2.04 ± 0.08	1.89 ± 0.04	2.91 ± 0.09	2.97 ± 0.06	3.76 ± 0.35	4.3 ± 0.55
D‐Limonene	14.68 ± 0.51	14.69 ± 0.56	13.31 ± 0.54	9.15 ± 1.48	17.29 ± 1.01	18.83 ± 0.42	20.86 ± 2.50	22.14 ± 0.93
ß‐Phellandrene	5.37 ± 0.23	4.79 ± 0.01	5.15 ± 0.10	3.81 ± 0.01	9.56 ± 0.47	9.75 ± 0.32	8.55 ± 0.22	9.38 ± 0.56
1,3,6‐Octatriene, 3,7‐dimethyl‐, (Z)—	1.19 ± 0.02	1.21 ± 0.04	1.08 ± 0.01	1.09 ± 0.01	0.42 ± 0.05	0.37 ± 0.04	0.57 ± 0.06	0.51 ± 0.03
β‐Terpinene	2.06 ± 0.01	2.10 ± 0.05	1.93 ± 0.02	1.77 ± 0.03	1.01 ± 0.04	0.88 ± 0.04	0.91 ± 0.03	0.73 ± 0.02
2,4,6‐Octatriene, 2,6‐dimethyl‐, (E, Z)—	0.36 ± 0.02	0.29 ± 0.02	0.22 ± 0.01	0.21 ± 0.01	ND	ND	ND	ND
Caryophyllene	3.19 ± 0.26	3.68 ± 0.24	2.40 ± 0.04	2.99 ± 0.05	2.29 ± 0.08	2.63 ± 0.40	3.62 ± 0.43	4.5 ± 0.30
1,3‐Cyclohexadiene, 5‐(1,5‐dimethyl‐4‐hexenyl)‐2‐methyl—	0.52 ± 0.04	0.28 ± 0.05	0.36 ± 0.03	0.26 ± 0.02	ND	ND	ND	ND
3‐Vinyl‐1,2‐dithiacyclohex‐4‐ene	1.81 ± 0.07	1.81 ± 0.20	1.98 ± 0.05	2.44 ± 0.06	0.73 ± 0.04	0.81 ± 0.07	0.87 ± 0.06	1.16 ± 0.13
Subtotal	39.00 ± 1.45	37.87 ± 0.69	35.23 ± 0.60	28.88 ± 1.32	43.73 ± 1.48	45.82 ± 1.01	47.82 ± 3.52	52.83 ± 1.80
**Esters**								
4‐Terpinenyl acetate	1.47 ± 0.05	1.14 ± 0.07	1.25 ± 0.01	1.07 ± 0.04	1.64 ± 0.20	1.98 ± 0.31	1.55 ± 0.16	1.31 ± 0.22
Linalyl acetate	24.74 ± 1.45	23.34 ± 2.18	24.12 ± 0.53	20.14 ± 1.95	15.22 ± 0.84	13.48 ± 1.18	9.44 ± 0.73	19.57 ± 1.22
α‐Terpinyl acetate	0.99 ± 0.05	0.78 ± 0.12	0.73 ± 0.10	0.81 ± 0.03	0.42 ± 0.04	0.52 ± 0.03	0.47 ± 0.05	0.52 ± 0.04
Subtotal	27.2 ± 1.45	25.25 ± 2.33	26.1 ± 0.43	22.02 ± 1.89	17.28 ± 0.68	15.98 ± 0.88	11.46 ± 0.84	21.40 ± 1.47
**Aldehydes**								
Nonanal	1.08 ± 0.04	0.87 ± 0.06	1.44 ± 0.07	1.03 ± 0.01	0.61 ± 0.08	0.52 ± 0.03	0.55 ± 0.05	0.62 ± 0.03
Subtotal	1.08 ± 0.04	0.87 ± 0.06	1.44 ± 0.07	1.03 ± 0.01	0.61 ± 0.08	0.52 ± 0.03	0.55 ± 0.05	0.62 ± 0.03
**Ketones**								
5‐Hepten‐2‐one, 6‐methyl—	1.10 ± 0.12	0.93 ± 0.01	1.24 ± 0.18	1.10 ± 0.02	0.79 ± 0.03	0.72 ± 0.05	0.84 ± 0.05	1.20 ± 0.06
Subtotal	1.10 ± 0.12	0.93 ± 0.01	1.24 ± 0.18	1.10 ± 0.02	0.79 ± 0.03	0.72 ± 0.05	0.84 ± 0.05	1.20 ± 0.06
**Aromatic hydrocarbon**								
Benzene, 1‐methyl‐2‐(1‐methylethyl)—	1.36 ± 0.11	1.17 ± 0.02	1.40 ± 0.03	1.17 ± 0.04	0.43 ± 0.04	0.39 ± 0.03	0.62 ± 0.04	0.37 ± 0.03
Benzene, 1,3‐bis (1,1‐dimethylethyl)—	ND	ND	0.87 ± 0.19	0.68 ± 0.02	ND	ND	1.56 ± 0.12	1.33 ± 0.11
Subtotal	1.36 ± 0.11	1.17 ± 0.02	2.2 ± 70.16	1.85 ± 0.02	0.43 ± 0.04	0.39 ± 0.03	2.18 ± 0.08	1.70 ± 0.14
**Furans**								
2‐n‐Pentylfuran	2.72 ± 0.06	2.73 ± 0.10	2.40 ± 0.11	2.64 ± 0.30	ND	ND	ND	ND
Subtotal	2.72 ± 0.06	2.73 ± 0.10	2.40 ± 0.11	2.64 ± 0.30	ND	ND	ND	ND
**Sulfur compounds**								
Diallyl disulphide	3.31 ± 0.14	3.12 ± 0.18	4.20 ± 0.08	4.87 ± 0.25	3.61 ± 0.04	2.81 ± 0.27	1.58 ± 0.11	1.85 ± 0.06
(E)‐1‐Allyl‐2‐(prop‐1‐en‐1‐yl)disulfane	0.22 ± 0.03	0.20 ± 0.03	0.33 ± 0.02	0.80 ± 0.11	ND	ND	ND	ND
Subtotal	3.53 ± 0.12	3.32 ± 0.2	4.52 ± 0.10	5.67 ± 0.17	3.61 ± 0.04	2.81 ± 0.27	1.58 ± 0.11	1.85 ± 0.06
**Others**								
Geranyl vinyl ether	0.49 ± 0.06	0.34 ± 0.04	0.49 ± 0.09	0.45 ± 0.01	ND	ND	ND	ND
Subtotal	0.49 ± 0.06	0.34 ± 0.04	0.49 ± 0.09	0.45 ± 0.01	ND	ND	ND	ND

Abbreviation: ND, not detected.

## 4 Conclusion

The results of the present study indicated that gamma irradiation combined with four kinds of antioxidants (α‐tocopherol, phytic acid, tea polyphenols, and tertiary butydroquinone) maintained the color and POV compared with samples irradiated at 4 kGy without antioxidants. Adding antioxidants inhibited the degradation of monounsaturated fatty acids and polyunsaturated fatty acids caused by irradiation. Changes in odor caused by irradiation with antioxidants were smaller than those caused by irradiation without antioxidants. Antioxidants promote the accumulation of alkenes. Therefore, we consider that 4 kGy irradiation with four kinds of antioxidants can not only prolong the shelf life of spicy chicken meat but also inhibit the negative effects of irradiation, which is conducive to maintaining the quality of meat.

## Conflicts of Interest

The authors declare no conflicts of interest.

## Data Availability

The data that support the findings of this study are available on request from the corresponding author. The data are not publicly available due to privacy or ethical restrictions.
